# Combination effect of flavonoids attenuates lung cancer cell proliferation by inhibiting the STAT3 and FAK signaling pathway

**DOI:** 10.1515/biol-2022-0977

**Published:** 2024-11-22

**Authors:** Pei Wan, Xingru Li, Shiqi Guo, Xiangling Zhao

**Affiliations:** Department of Thoracic and Cardiovascular Surgery, The First College of Clinical Medical Science, China Three Gorges University, Yichang Central People’s Hospital, Yichang, Hubei, 443000, China; Department of Respiratory and Critical Care Medicine, Lu’an People’s Hospital of Anhui Province, Lu’an, Anhui, 237005, China

**Keywords:** delphinidin, oroxylin A, cell proliferation, STAT3 and FAK, lung cancer

## Abstract

Lung cancer is considered the most ubiquitous malignant form of cancer, and the current treatment strategies do not offer effective outcomes to the patients. The present study examined the effectiveness of natural drugs delphinidin (DN) and oroxylin A (OA) in inhibiting the development of lung cancer cells (A549) through the blocking of the Signal transducer and activator of transcription 3 (STAT3) and focal adhesion kinase (FAK) intervene signaling pathways. These included cytotoxicity assessments, reactive oxygen species (ROS) levels, apoptotic morphological features, mitochondrial membrane potential (ΔΨm), nuclear fragmentation, and cell cycle analysis. Furthermore, the combination of DN and OA treatments on the expression of STAT-3, FAK, and various proliferation and apoptotic proteins was studied using western blotting. The results we have obtained are that the combination of DN and OA causes significant cytotoxicity, ROS, alteration of ΔΨm, and nuclear fragmentation, resulting in apoptosis of A549 cells. Furthermore, A549 cells treated with DN and OA concurrently displayed increased cell cycle arrest at the G2/M phase. Additionally, the combined DN and OA treatment inhibited the expression of STAT3 and FAK, suppressing proliferation and the induction of pro-apoptotic protein expressions in A549 cells. Thus, a combination of DN and OA could be used as a therapeutical approach to malignant forms of lung cancer.

## 1 Introduction

Cancer is typically seen as a deadly disorder that poses a significant threat to public health. It is characterized by unchecked development and proliferation of cells brought on by the accumulation of hereditary alterations [1,2,3]. Lung cancer is considered as the most prevalent type of malignancy, predicted to have 2.2 million newly diagnosed instances and 1.8 million fatalities globally during the year 2020 [4]. It has been identified as the main factor in both morbidity and mortality from cancer in males. The disease is the second leading cause of death in females and the third leading cause of morbidity. According to Tung et al. [5], non-small cell lung cancer (NSCLC) represents about 85–88% of all instances of lung cancer while small cell lung cancer (SCLC) represents 12–15% [5]. Given the steadily rising rate of malignant diseases around the world, the discovery of innovative and effective treatments for cancer prevention is crucial. By 2040, there will be 28.4 million additional cases of cancer worldwide, representing an estimated 50% increase over the following two decades [4]. Currently, diagnosis of lung cancer has been followed by traditional and machine learning techniques such as automated detection systems to confirm the early stages of lung cancer [6]. Traditional cancer treatments have a number of unfavorable side effects, including intolerance, drugs resistance, and detrimental effects on healthy tissue. Hence, considering their significant therapeutic benefits, which include fewer side effects, improved health, and the ability to treat tumors; plant-derived natural products are regarded as safe therapy possibilities.

Lung cancer cell proliferation is driven by numerous cell signaling events. Signal transducer and activator of transcription 3 (STAT-3) is believed to be a key transcription factor that, once phosphorylated, translocate to the nucleus and promote the expression of various proliferative genes [7]. Similarly, focal adhesion kinase (FAK), a member of the tyrosine kinase family, is often overexpressed in tumor cells and plays a crucial role in promoting malignant transformation by enhancing cell survival and proliferation [8]. Inhibiting STAT3 and FAK signaling pathways is considered a promising strategy for treating lung cancer. However, the use of single drugs is often compromised by drug-detoxifying enzymes and multidrug resistance proteins. As a result, combination therapy is seen as a more effective approach, enhancing therapeutic efficacy and potential [9].

Based on their appealing coloring characteristics and possible beneficial effects on health, anthocyanins, a category of flavonoids, are consumed in fruits and vegetables, primarily in berries, grapes, pomegranates, red cabbage, and sweet potatoes, as well as red wine [10,11]. They also rank among the most frequently used natural food dietary supplements [12]. One of the most well-known anthocyanidins (aglycons of anthocyanins) is delphinidin (DN). As when compared with various flavonoid molecules, DN has a higher oxidative biological action since it has three hydroxyl groups on its B ring. Investigations have found more and more instances where DN may be able to prevent carcinogenesis [13]. DN has a cancer-preventing impact, although its precise mechanism remains undetermined. To find more potent anti-tumor drugs derived from the natural chemical, modifying the molecular structure of DN was essential. Oroxylin A (OA), a type of flavonoid obtained from Scutellariae radix, prevents the development of Tregs in NSCLC. OA was frequently employed to treat bacterial, viral, and viral-related illnesses, as well as inflammation and cancer [19]. Appropriate doses of OA exhibit no harm to healthy cells and tissues, indicating a benefit over conventional chemotherapeutic medicines. Nevertheless, no scientific proof exists to prove that combining DN and OA, especially in target-specific pathways, has an anticancer impact on lung cancer. We demonstrate here that both the natural drugs combo DN and OA suppressed lung cancer cell development and caused cell death. The current study aimed to determine how effectively lung cancer cells (A549) might be prevented from growing by blocking the STAT3 and FAK intervention signaling pathways. The synergistic actions of OA and DN, two natural medicines, were utilized to achieve this. As a result, DN and OA may be viable new therapeutic agents for lung cancer treatment.

## 2 Material methods

### 2.1 Cell culture and maintenance of lung cancer cells

Human lung cancer cells (A549) were grown in DMEM medium, supplemented with 5% fetal bovine, 1% streptomycin/penicillin, and an incubator set at 37°C with 5% CO_2_. Following an 80% cell density achievement in a 175 cm^2^ flask, the cells were cultivated using trypsin-EDTA and washed with phosphate-buffered saline (PBS; pH 7.4). The culture media was updated approximately every 2–3 days.

### 2.2 *In vitro* cytotoxicity study

Initially, the cytotoxicity of DN, OA, and DN + OA was assessed. Specifically, 96-well plates were used to cultivate A549 cells (5 × 10^3^ cells/well), which were then incubated for 24 h. They then had DN, OA, and DN + OA therapy for 24 h. After that, each plate was incubated for 4 h at 37°C with 20 µL of MTT added to each well. The formazan crystals were removed from the MTT-containing solution and put into each well. 150 µL of DMSO was then used to liquefy them. After that, the plates were shaken for 10 min at 37°C. The 550 nm absorbance of the colored solution was then determined using a microplate reader. The following formula was used to determine the cell viability:

\[ \% \hspace{.25em}\text{cell}\hspace{.25em}\text{viability}=(\text{OD}\hspace{.25em}\text{value}\hspace{.25em}\text{of}\hspace{.25em}\text{test}/\text{OD}\hspace{.25em}\text{value}\hspace{.25em}\text{of}\hspace{.25em}\text{control})\times 100.]\]

### 2.3 Evaluation of drug combinations

A constant ratio based on the compound’s IC_50_ values was applied to cells (1 million cells/well) to assess any potential synergistic effects of DN, OA, and DN + OA. Using the combination index (CI), as defined by Chou and Talalay [14], the cytotoxicity of the combination was compared to the cytotoxicity of every compound separately. The compounds function synergistically if the CI is less than 1, additively if the CI is equal to 1, and antagonistically if the CI is greater than 1. Since lower values are not considered significant for preventing growth, the mean CI was computed using the DN, OA, and DN + OA > 0.5 data points. The concentrations of DN + OA employed in the tests were 10, 20, and 30 μM.

### 2.4 Morphological characterization of cell nuclei using 4′,6-diamidino-2-phenylindole (DAPI) staining

Using DAPI labeling, it was possible to observe the apoptotic body generation and nuclear alterations indicative of that cell death. After assigning with DAPI (1 μg/mL), cells were treated with DN and OA for 24 h. Phase contrast and fluorescence microscopy were used to evaluate any cell morphological alterations. DAPI labeling identified the DN, OA, and DN + OA for 24 h, with untreated cells serving as controls. Fluorometric observations were made of DAPI-stained cells for 15 min, using emission at 358 nm and excitation at 461 nm.

Dual staining with AO/EtBr In A549 cells treated with DN, OA, or a combination of DN and OA, the proportion of cell apoptosis was ascertained using AO/EtBr staining. A549 cells were plated (1 × 10^4^/well) and incubated at 37°C for 24 h. Following DN and OA therapy, the cells were incubated for 24 h at 37°C. The treated cells were stained for 10 min with 100 µg/mL of AO/EtBr dye in a 1:1 ratio after the treatment stage. The amount of apoptotic cell death in the treated cells was quantified using a fluorescent microscope (3501, Lawrence and Mayo India Pvt Ltd).

### 2.5 Mitochondrial membrane potential assay (ΔΨM)

Employing Rh-123 staining techniques, the amount of mitochondrial membrane potential (ΔΨM) in A549 cells exposed to DN, OA, and DN + OA was assessed. To accomplish this, 1 × 10^4^ A549 cells were inserted into every well of a 24-well plate, and the cells were then incubated at 37°C for 24 h. After that, the cells were treated with DN, OA, and DN + OA for 24 h at 37°C. Following 30 min of Rh-123 staining the cells at a dose of 10 µg/mL, the fluorescence brightness was measured with a fluorescence microscope.

### 2.6 Intracellular generation of reactive oxygen species (ROS)

The ROS generation experiment was conducted following previous guidelines [15]. The A549 cells were treated for 24 h with DN, OA, and DN + OA. Afterward, they were washed with PBS and reconstituted in culture media (separate from serum) with 10 µM 2′,7′-dichlorofluorescein diacetate (DCFH-DA). Following that, ROS production was determined by flow cytometry.

### 2.7 Apoptosis detection via flow cytometry

Annexin V-fluorescein isothiocyanate (V-FITC) cell apoptosis detection kits (Beyotime, Shanghai, China) were used to recognize apoptosis. In each well, 1 × 10^4^ A549 cells were seeded, and those groups undergoing treatment (DN, OA, and DN + OA with medium supplementation) were incubated for 24 h at 37°C. The cells were incorporated in a binding buffer after being cultured for 24 h at the proper DN, OA, and DN + OA concentrations. The cells were exposed to air for 20 min after being stained with 5 μL of Annexin V-fluorescein isothiocyanate (FITC) and 10 μL of phenylindole (PI). With the use of flow cytometry, every cell was gathered (BD Biosciences, San Jose, CA, USA) in less than an hour.

### 2.8 Cell cycle arrest analysis

DN, OA, and DN + OA were added to A549 cells (1 × 10^4^) in culture for 24 h. The cells were removed and preserved for 12 h at 4°C in ice-cold 70% ethanol. The sample was immersed again in a solution containing 25 μL PI after being cleaned with PBS. 10 μL of RNase A was then dyed in buffer (0.5 mL) and allowed to sit in the dark at 37°C for half an hour. Then, the cell cycle’s phase distribution was found using a flow cytometer.

### 2.9 Western blot analysis

Employing Western blotting, DN, OA, and the combined impact of DN + OA on the apoptotic and anti-apoptotic proteins in the treatment and control cells were studied. The selected DN and OA drug formulations were applied to A549 cells for 24 h in a 100 mm culture dish. The cells underwent many PBS washes after the medium was taken out. Following a 10 min incubation period in lysis buffer, the cells were harvested, subjected to a 30 min shaking, and centrifuged at 12,000*g* for 10 min at 4°C. Using a bicinchoninic acid protein test kit, the protein content was measured after the supernatant was gathered. Protein samples were electrophoretically loaded onto a 10% polyacrylamide gel and moved onto a PVDF membrane. The membrane was treated with certain antibodies after being blocked in tris-buffered saline with Tween 20, including 5% skim milk. After an overnight period at 4°C, the primary antibodies were washed out, and the secondary antibodies were added. At room temperature, the protein bands became visible after an hour of incubation. Ultimately, the membrane was found using BeyoECL Plus from Beyotime.

### 2.10 Statistics

Every experiment was run over three times separately, and the entire data are presented as mean value ± standard deviation (SD). To do a statistical evaluation, GraphPad Prism 6.0 was employed. For several group contrasts, a one-way analysis of variance was applied, and Tukey’s *post hoc* test was accompanied. In statistical terms, the symbol * denotes the degree of significance concerning the control, and *P* values are shown as *P* values <0.05 being considered significant.

## 3 Results

### 3.1 Impact of DN and OA therapy on the A549 cell line’s viability

**Figure 1:**
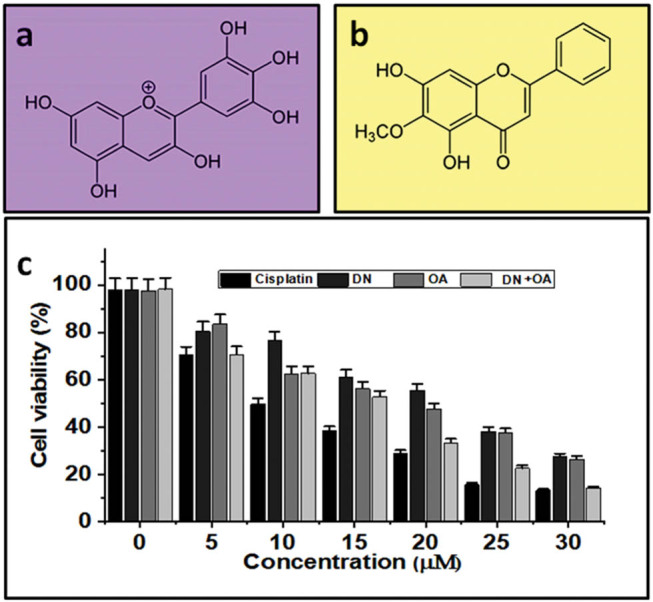
Chemical structure of (a) DN and (b) OA. The proliferation of A549 lung cancer cell lines was suppressed by the combined use of DN and OA. (c) For 24 h, A549 cells were treated with DN and OA at the specified concentrations. The MTT assay was used to evaluate cell viability.

Specifically, we present a comprehensive experimental analysis of DN, OA, and their combination’s anticancer activity against A549 cell lines. To evaluate the growth-inhibitory properties of DN and OA, both of their combined impacts on the growing activities of the cells were studied (Figure 1). Figure 1a and b depicts the DN and OA structures.

The experiment aimed to investigate how DN and OA interacted in A549 lung cancer cells. DN and OA were tested separately and in combo for their impact on lung cancer cell viability using the MTT (3-(4,5-dimethylthiazol-2-yl)-2,5-diphenyltetrazolium bromide) assay. As seen in Figure 1c, the simultaneous administration of DN and OA dramatically reduced the viability of the A549 cell lines in a dose-varying way (*P* < 0.01). These findings show that OA and DN are potent therapies for lung cancer cells. It was also found that this effect increased with the use of drug combos. Table 1 and Figure 1c shows the percentage of cell viability and IC50 concentration of DN, OA, DN + OA and Cisplatin. Figure 1 shows that there was no discernible variation in the viability of the cells treated with the substance and those left untreated.

**Table 1:** IC_50_ concentrations of DN, OA, DN + OA, and cisplatin

Compounds	IC_50_ concentration (µM)
DN	21.6 ± 0.4
OA	19.12 ± 0.3
DN + OA	16.3 ± 0.7
Cisplatin	10.6 ± 0.2

### 3.2 Assessment of drug interactions in A549 cells

The CI was calculated using the CompuSyn program. It proved that the amounts of 10, 20, and 30 µM DN and OA simultaneously diminished cellular viability in lung cancer cells. The CI values connected to some of the combinations are displayed in Table 2. A CI value of more than 1 indicates that the two compounds are antagonistic to one another. The values of CI = 1:1 indicates that the two components work in concert. In A549 cells, the CI value at 10 µM DN and 10 µM OA is almost synergistic (CI 0.56); at 30 µM DN and 20 µM OA, on the other hand, the CI values demonstrate great synergism (CI 0.59). To enhance our comprehension of their impacts at the cellular level, we decided to persist with the subsequent ratios (1). Each at 10 µM for DN and OA; (2). At 20 µM, DN and OA; (3). OA and DN at 30 µM.

**Table 2:** CI values for DN and OA (1:1)

Concentrations (µM)	CI value	CI effect
10	0.56278	Synergistic
20	0.59762	Synergistic
30	0.63869	Synergistic
40	0.74635	Moderate synergistic
50	0.77235	Moderate synergistic

### 3.3 Combination of DN and OA on morphological assessment in A549 cells

**Figure 2:**
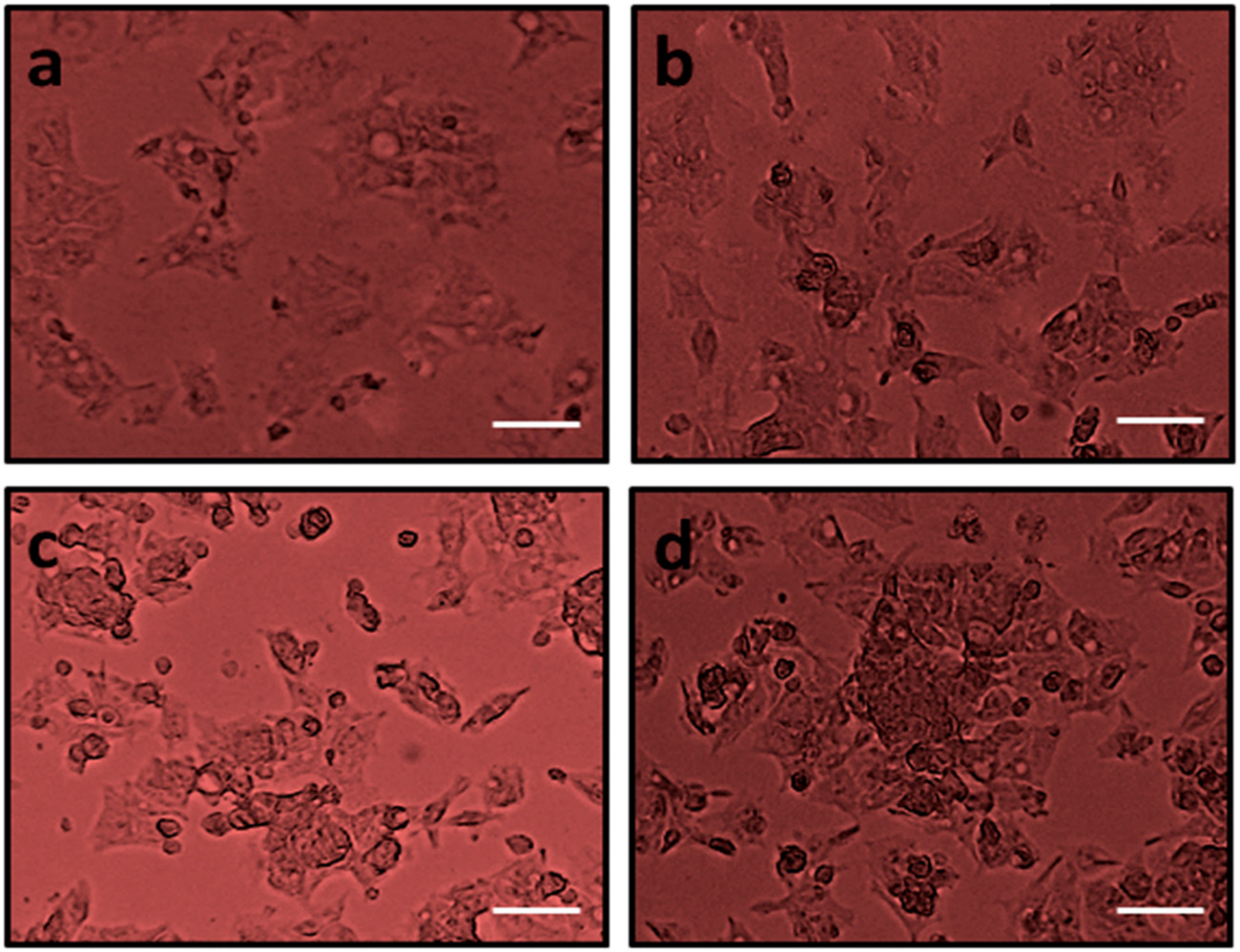
Cytotoxicity effect of DN and OA at different dosages on the A549 cells. The morphology changes absorbed from control and DN and OA at different dosages treated against A549 cancer cells in phase contrast microscopy (a) control, cells treated with (b) 10 µM DN and OA, (c) 20 µM PN and OA, and (d) 30 µM DN and OA. The scale bar displayed 50 µm.

Human lung cancer cells (A549) in growth were used to assess the damaging responses to DN + OA in addition to controls. Phase-contrast microscopy was used to observe that DN, OA alone, and in combination with DN, OA significantly triggered mortality in cancer cells. In contrast, untreated cells’ viability was slightly altered at 24 h (Figure 2). The DN, OA, and DN + OA-treated cells had altered, taking on a bow-like morphology and seemingly extensive damage.

### 3.4 Combination of DN and OA on cell migration in A549 cells

**Figure 3:**
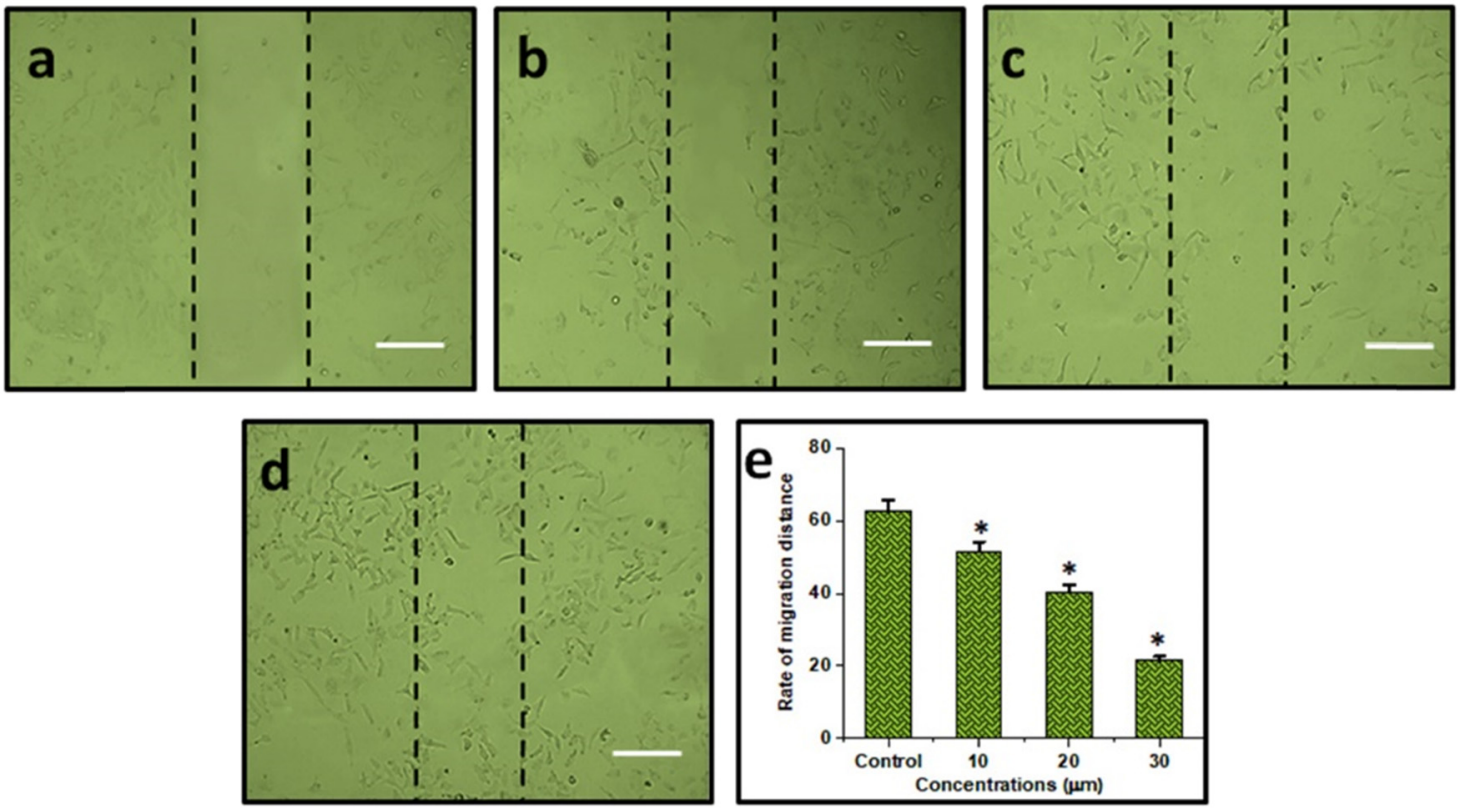
Cell migration assay: (a) control; (b) cells treated with 10 µM combination of DN + OA; (c) cells treated with 20 µM combination of DN + OA; (d) cells treated with 30 µM of DN + OA. (e) A statistical examination of the frequency of cell migration. The mean value ± SD is shown, *P < 0.05. The scale bar displayed 50 µm.

Cancer cells’ ability to survive, migrate, and invade their surroundings is greatly influenced by the FAK/STAT3 signaling mechanism. The current investigation assessed the regulatory effects of the combination of DN + OA on cell migration. After incubating A549 cells with a combo of DN + OA (10, 20, 30 µM) for 24 h, changes were seen in the cell’s ability to migrate; their rates of migration were 62.5, 51.6, 40.4, and 21.5%, respectively. These findings demonstrated that the DN + OA combination considerably reduced the A549 cells’ migration ability (Figure 3a–e).

### 3.5 Combination of DN and OA on significant production of ROS in A549 cells

**Figure 4:**
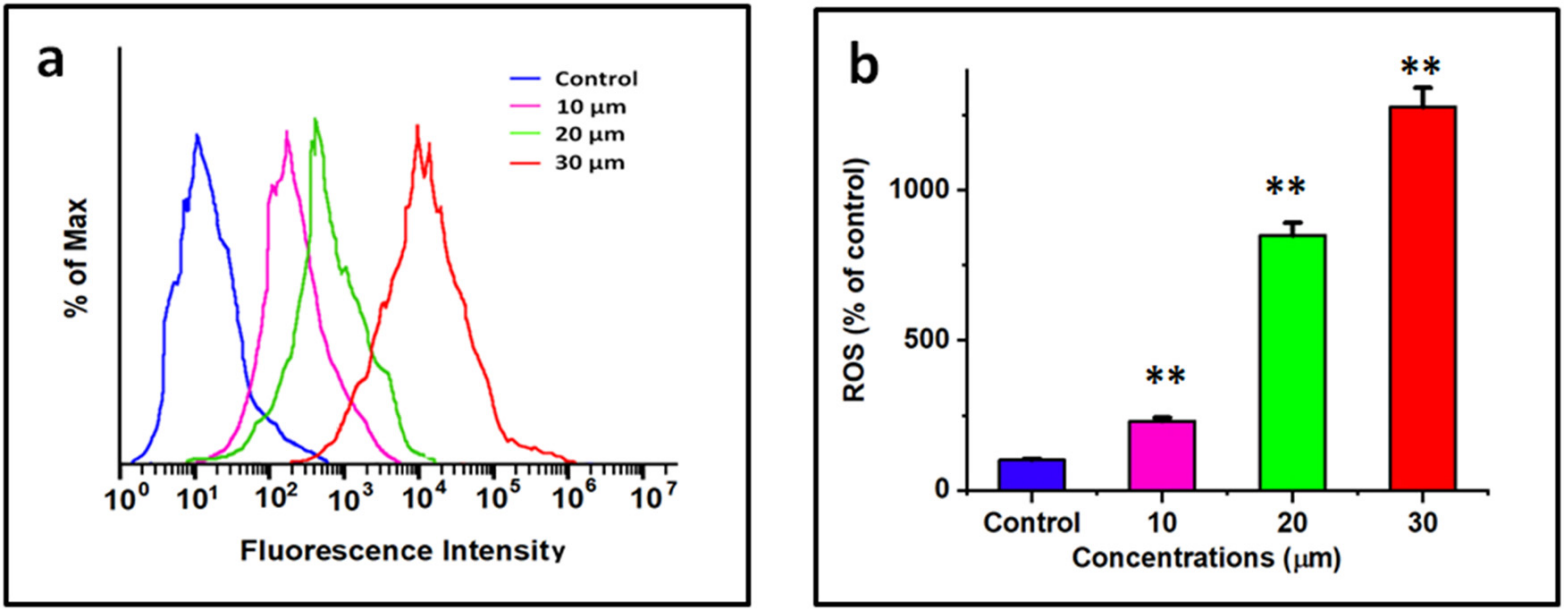
The method was feasible to investigate in A549 cells the impact of both DN and OA (10, 20, and 30 µM of DN + OA) on the production of intracellular ROS through the use of 2,7-dichlorofluorescein diacetate labeling. And flow cytometry (a) using a flow cytometer, the degree of intracellular ROS buildup was quantified and dyed with DCFH-DA. (b) Quantitative evaluation of intracellular ROS levels. The statistics represent the mean value and standard deviation of three duplicates with a **P < 0.05 significance level for comparing them to the controls group.

Using a DCFH-DA probe, the present study determined the intracellular level of formation of ROS in lung cancer cells, which was then validated by flow cytometry and confocal imaging. Following treatment with combined use of DN + OA for 24 h, the flow cytometry data demonstrated that the combination of DN + OA greatly increased ROS production (Figure 4a). Figure 6a illustrates the significant increase in green fluorescence value following a 24 h exposure to DN and OA at 30 µM/mL. The amount of fluorescence was evaluated using flow cytometry (Figure 4b), and quantitative evaluation of intracellular ROS levels was observed. After being treated simultaneously with DN and OA, A549 cells released stronger ROS, and this elevation was dose-dependent. These investigations brought into focus the lung cells’ innate apoptotic signaling pathways.

### 3.6 Combination of DN and OA on significant alteration matrix metalloproteinases (MMP) in A549 cells

**Figure 5:**
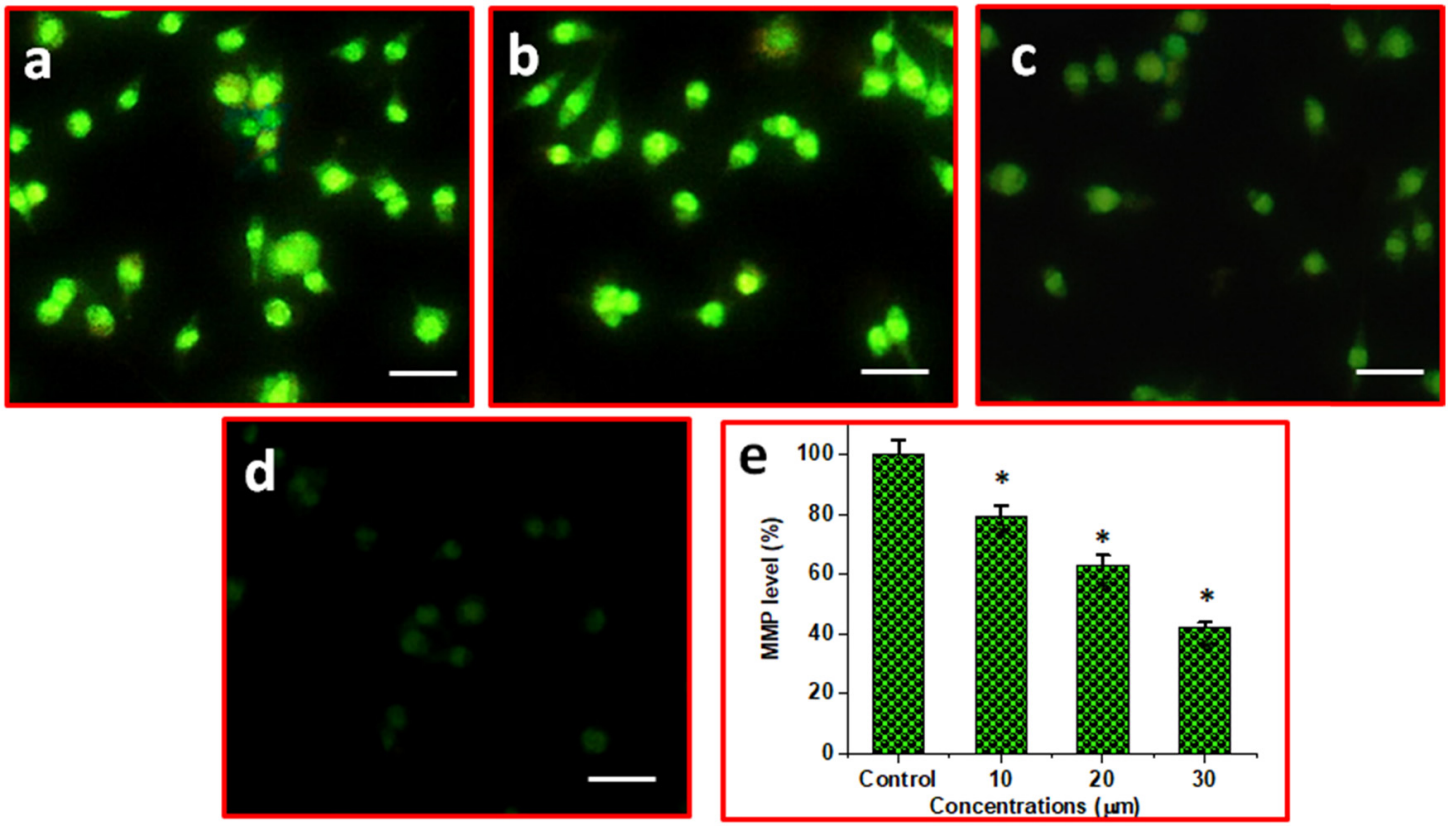
The impact of DN and OA combo on MMP levels in A549 cells. (a) Control, (b) 10 µM DN and OA, (c) 20 µM DN and OA, and (d) 30 µM DN and OA. (e) Bar illustration corresponds to the % MMP levels in control, combination of 10, 20, 30 µM DN and OA treated cells. The scale bar displayed 50 µm. *P < 0.05 significance level for comparing them to the controls group.

DN plus OA together cause A549 cells to exhibit apoptosis and increase MMP. A fluorescent microscope was utilized to identify the MMP modifications. The prevailing consensus is that MMP levels drop throughout apoptosis. The role of DN and OA therapy together was investigated employing rhodamine-123 staining, which is dependent on MMP modifications, an early marker of apoptotic properties. A549 cells were exposed to DN and OA (10, 20, and 30 µM) in varying doses throughout a 24 h, as shown in Figure 5. Initial indications of apoptosis were caused by the treatment and its effect on the MMP.

### 3.7 Combination of DN and OA on nuclear fragmentation in A549 cells

**Figure 6:**
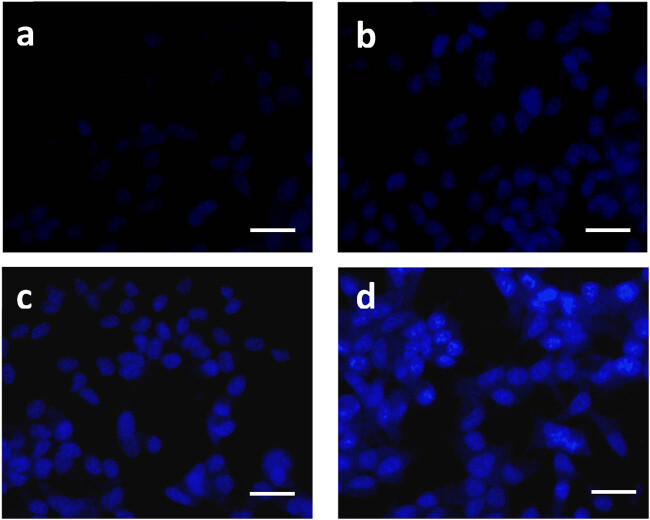
A549 cells with control nuclei labeled with DAPI and treated with DN and OA at different dosages. (a) Control, (b) 10 µM DN and OA, (c) 20 µM DN and OA, and (d) 30 µM DN and OA. Note that untreated cells have round nuclei and a less intense blue coloring. After exposure to both DN and OA, cells showed signs of apoptosis, including nuclear breakage and noticeably increased blue fluorescence light. The scale bar displayed 50 µm.

A549 cells exhibited apoptosis as revealed by DAPI staining, accompanied by chromatin condensate, apoptotic body, and cytoplasm and nuclear reduction. As demonstrated in Figure 6, DAPI-treated cells, or the untreated control, often exhibited blue color. Nonetheless, the groups treated with DN + OA showed a compact, compressed, and resembling appearance. The distinctive blue glow of the cells indicated that they were apoptotic. These outcomes correspond with the cytotoxicity data, showing that the synergistic action of DN + OA might trigger apoptosis against A549 cell lines.

### 3.8 Combination of DN and OA on apoptosis morphological features in A549 cells

**Figure 7:**
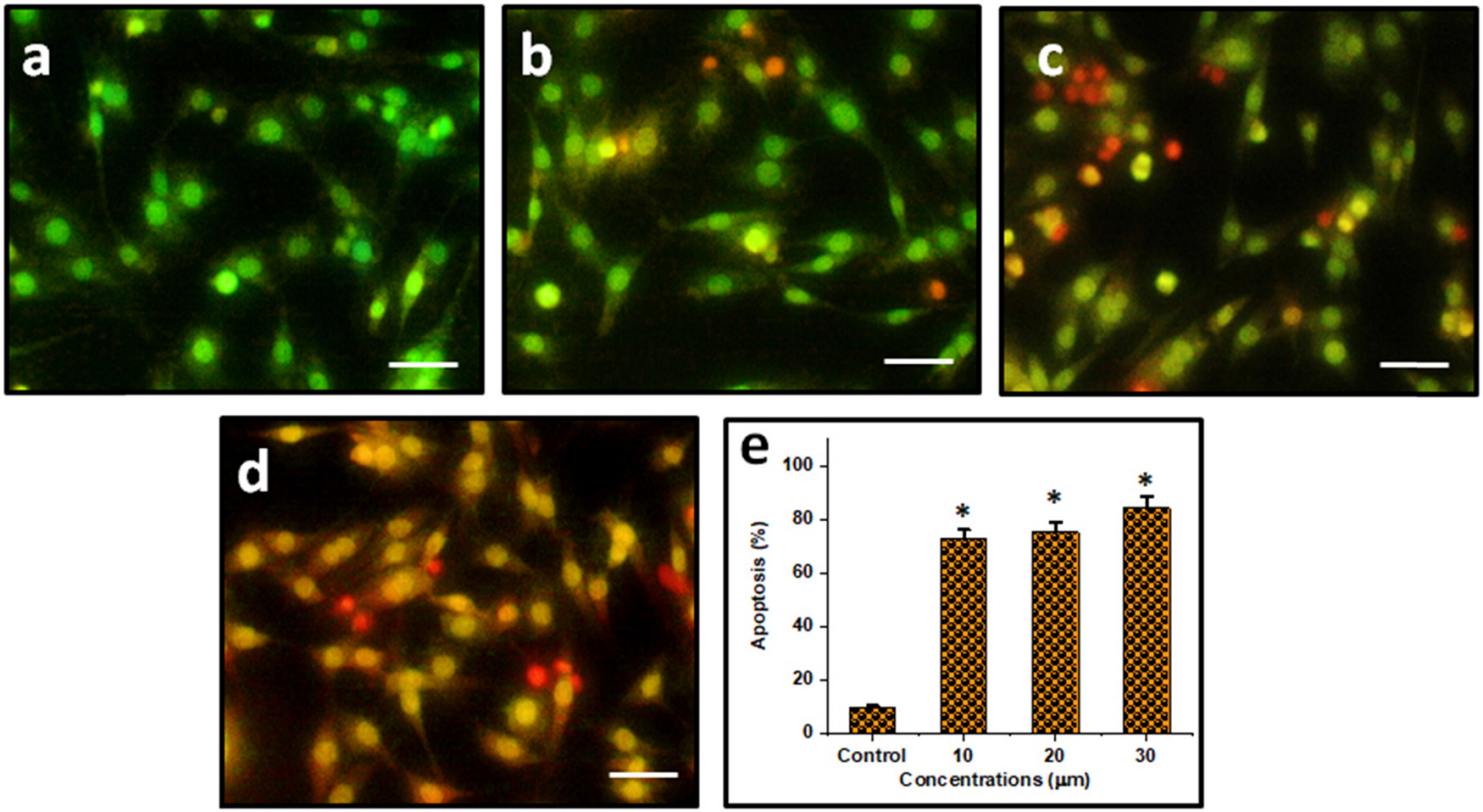
To attempt to determine the apoptotic stage using a combo of DN and OA, the cells were additionally stained with AO/EtBr. The investigation’s findings demonstrated that, according to a different hypothesis, a combination of DN and OA therapy protocol led to more robust apoptosis. (a) Control, (b) 10 µM DN and OA, (c) 20 µM DN and OA, and (d) 30 µM DN and OA. (e) Bar illustration corresponds to the % apoptosis in control, combination of 10, 20, 30 µM DN and OA treated cells. The scale bar displayed 50 µm.

Through the use of AO/EtBr labeling, we here show that the relationship among DN and OA affects the potential of lung cancer cells to enter apoptosis. Using a fluorescence microscope, AO/EtBr staining is a commonly used approach to observe chromatin alteration undergoing apoptosis. Fluorescence microscopy (Figure 7a–d) shows that the A549 cells’ apoptotic cells were treated with 10, 20, and 30 µM of DN and OA for a full day. The nucleus of the unaltered cell fluoresced green regularly. Conversely, fluorescence microscopy revealed that an impairment of the membrane’s stability was the cause of the uneven nucleus shape in the treated A549. In compositions where cells received both DN and OA therapy, reddish-orange structures known as apoptotic bodies which comprise nuclear constriction and bulging were observed. Consequently, the results of this investigation showed that, on a distinct premise, the combination of DN and OA treatment regimen provided stronger anti-tumor effects on lung cancer cells. Apoptotic cell proportions were 72.65, 75.18, and 84.33%, accordingly, at a range of dosages (10, 20, and 30 µM/mL) of the DN and OA treated categories (Figure 7e).

### 3.9 Combination of DN and OA on the function of the cell cycle in A549

**Figure 8:**
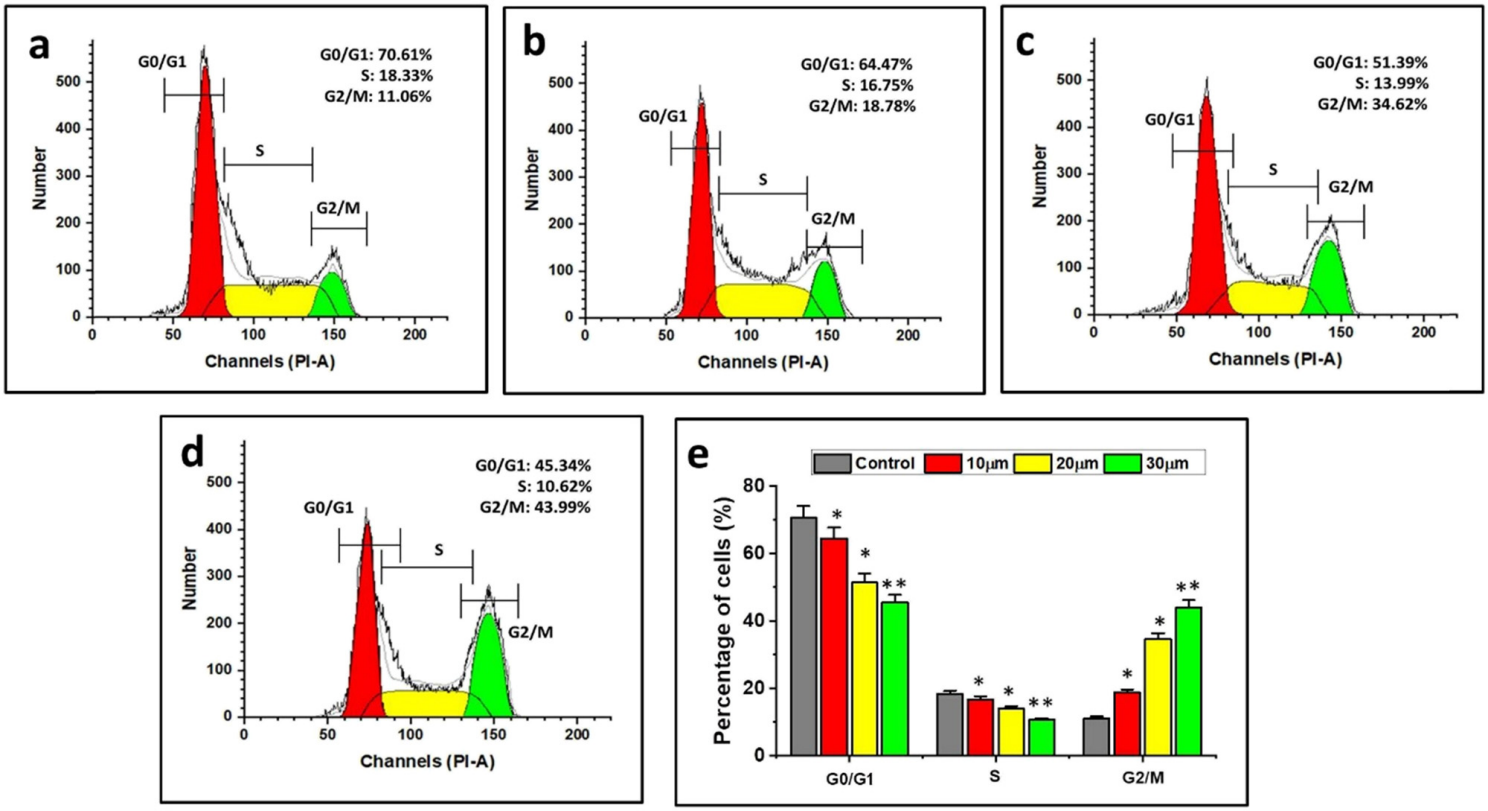
(a) Control, (b) 10 µM DN and OA, (c) 20 µM DN and OA, and (d) 30 µM DN and OA. According to the results of the cell cycle trials, co-treating A549 cells with DN and OA at varying doses caused the cells to terminate in the G2/M phase, indicating that the use of DN + OA together inhibited cell growth by arresting the cell cycle. (e) Statistical evaluation of assays for cell apoptosis. The data are shown as mean value ± SE. Mean significant difference (*P < 0.05, **P < 0.01) compared with the control.

The advancement of the cell cycle is crucial in controlling the proliferation of cancer cells. The aim of this experiment is to control the degree to which DN and OA together impeded the advancement of the cell cycle. The findings of the cell cycle analysis performed on the treated cells are shown in Figure 8. DN and OA (10, 20, 30 µM/mL) were incubated for 24 h in A549 cells, resulting in sub-G1 populations of 45.39%, S populations of 10.62, and G2/M populations of 43.99%, in that order. Most likely, these compounds caused the cell cycle arrest in A549 cells because the combined DN + OA treated cells were seen in the G2/M phase in contrast to the control cells. The outcomes of the cell cycle experiments showed that co-treating A549 cells with DN and OA at different doses resulted in an arrest in the G2/M phase of the cell, suggesting that the combined application of DN + OA hindered cell growth by halting the cell cycle (Figure 8).

### 3.10 Combination of DN and OA drugs suppressed function of STAT3

**Figure 9:**
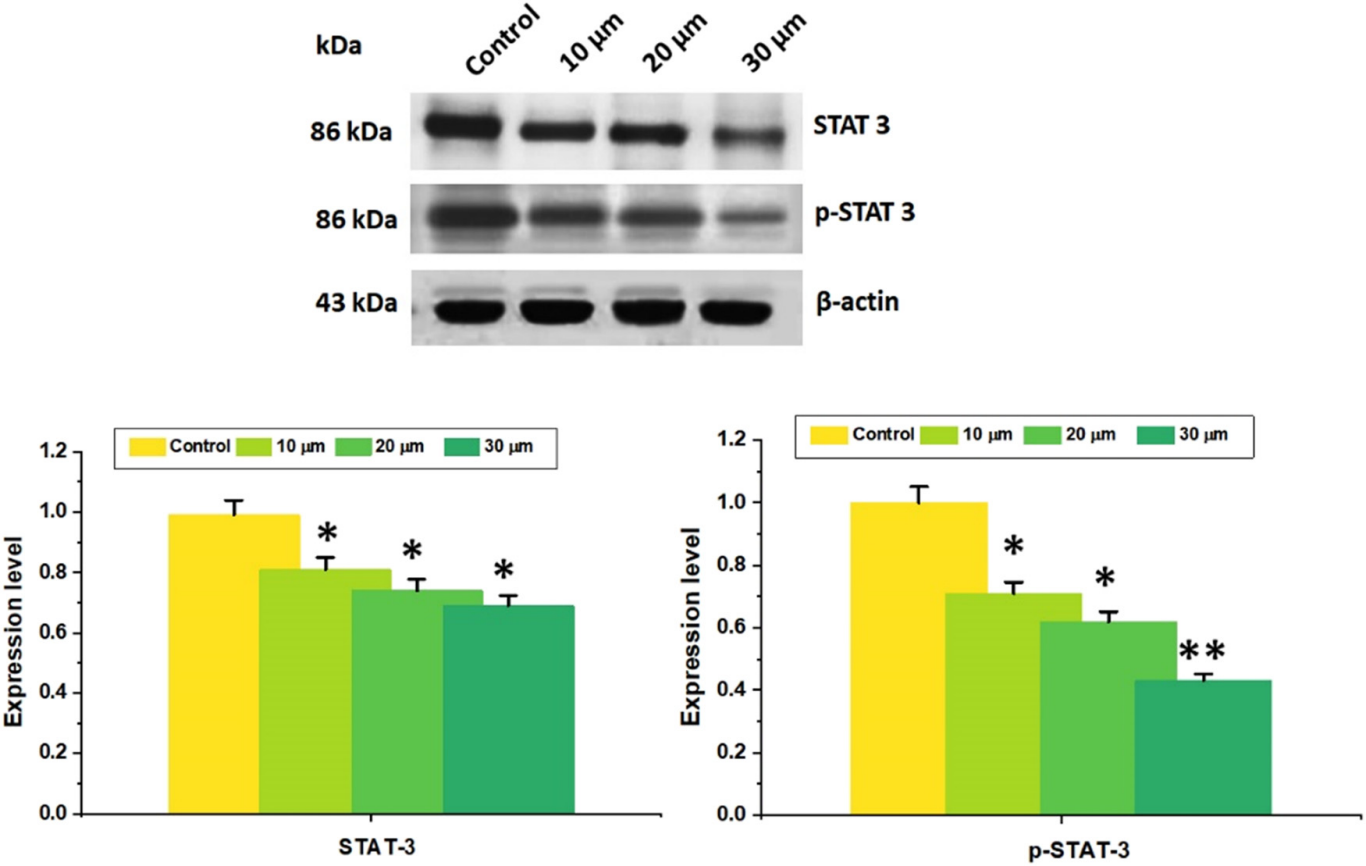
In A549 cells, combination treatment with DN + OA inhibited the constitutive activation of STAT3. STAT3 and p-STAT3 were found using western blotting (Tyr705). An internal control was employed, namely, β-Actin. The graphical representation of band intensities measured using Image J software analysis. The mean and standard deviation of three separate investigations were assessed. The mean value ± SD is shown, *P < 0.05, **P < 0.001.

Lung carcinoma cells have aberrant STAT3 activation, intimately linked to malignancies’ development and dissemination. Consequently, more research was done on DN, OA, and the combined impact of DN and OA to control constitutive STAT3 activity. Western blotting results revealed that when given a combo of DN and OA for 24 h, phosphorylated STAT3 activity declined while STAT3 levels remained unaffected. The findings showed that in A549 cells, the combination of DN and OA inhibited the intrinsic phosphorylation of STAT3 (Figure 9a and b). All things considered, DN with OA was found to be a potential STAT3 suppressor.

### 3.11 Combined effect of DN and OA suppressed cell growth by specifically targeting STAT3

**Figure 10:**
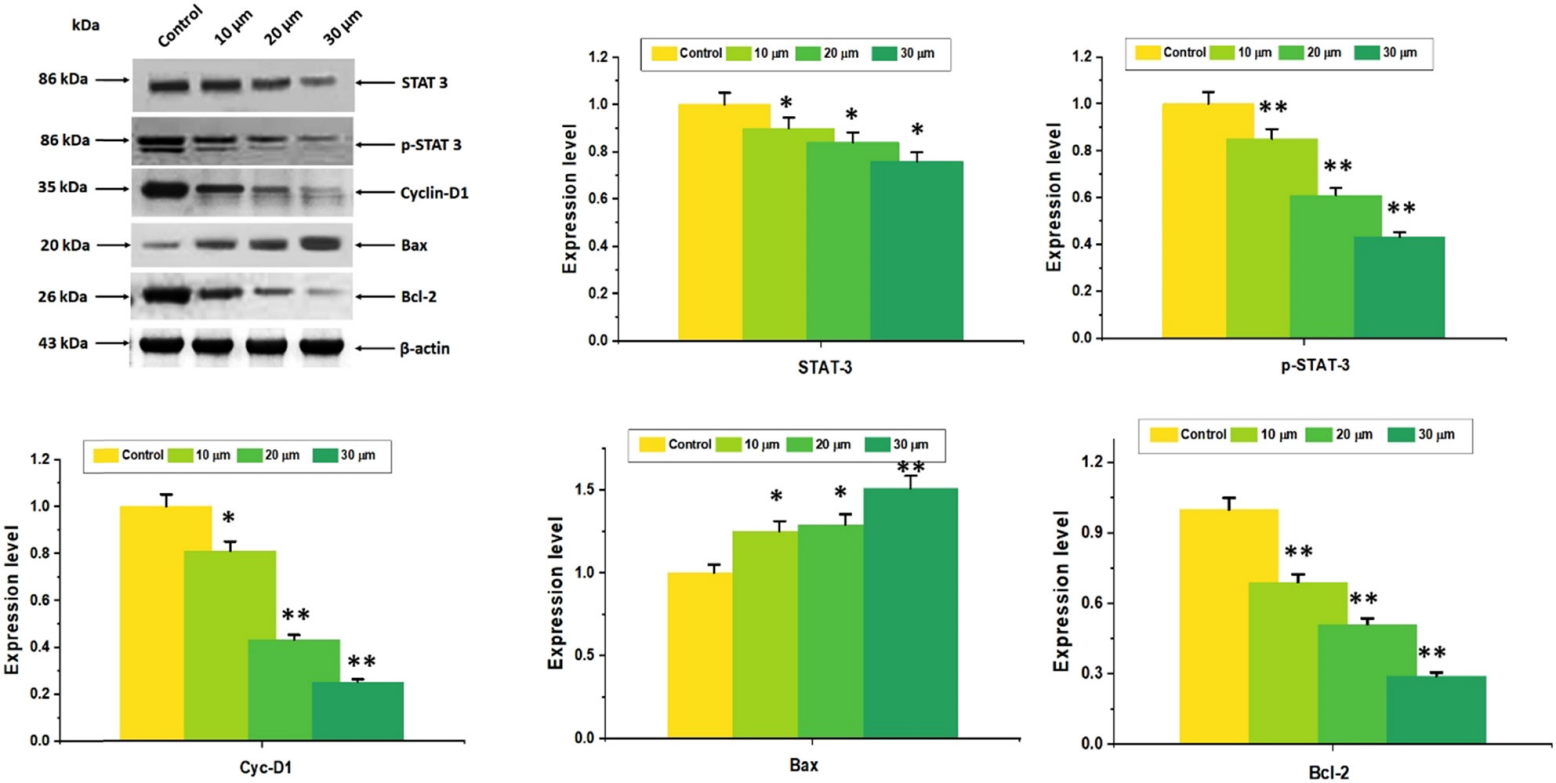
The combination DN + OA directly targeted STAT3 in A549 cells to decrease cell growth. Several proteins (STAT3, p-STAT3, cyclin-D1, Bcl-2, Bax) were measured using Western blotting analysis. The densitometric assessment performed earlier indicated that proteins were assessed using the Image Studio software. The replication of the housekeeping gene, β-actin, verified the equal loading of proteins. The mean value as well as the standard deviation of three separate experiments were assessed. The mean value ± SD is shown, *P < 0.05, **P < 0.001.

Initially, the combined impacts of OA and DN prevented STAT3 from being phosphorylated; additional investigation was required to determine how both combinations of DN + OA influenced the expression of downstream STAT3 signaling cascade components. A crucial protein in the formation of the cell cycle is cyclin D1. Western blotting revealed that in cells exposed to a combination of DN + OA, there was a substantial reduction in the expression levels of cyclin D1. After receiving a combination of DN and OA, it was discovered that the level of expression of the anti-apoptotic protein Bcl-2 decreased. In the DN + OA treated study, there was a concurrent rise in the expression of the pro-apoptotic protein Bax (Figure 10a and b). The findings verify the strong relationship between STAT3 and tumor cell death.

### 3.12 Combined DN and OA inhibited the FAK signaling pathway

**Figure 11:**
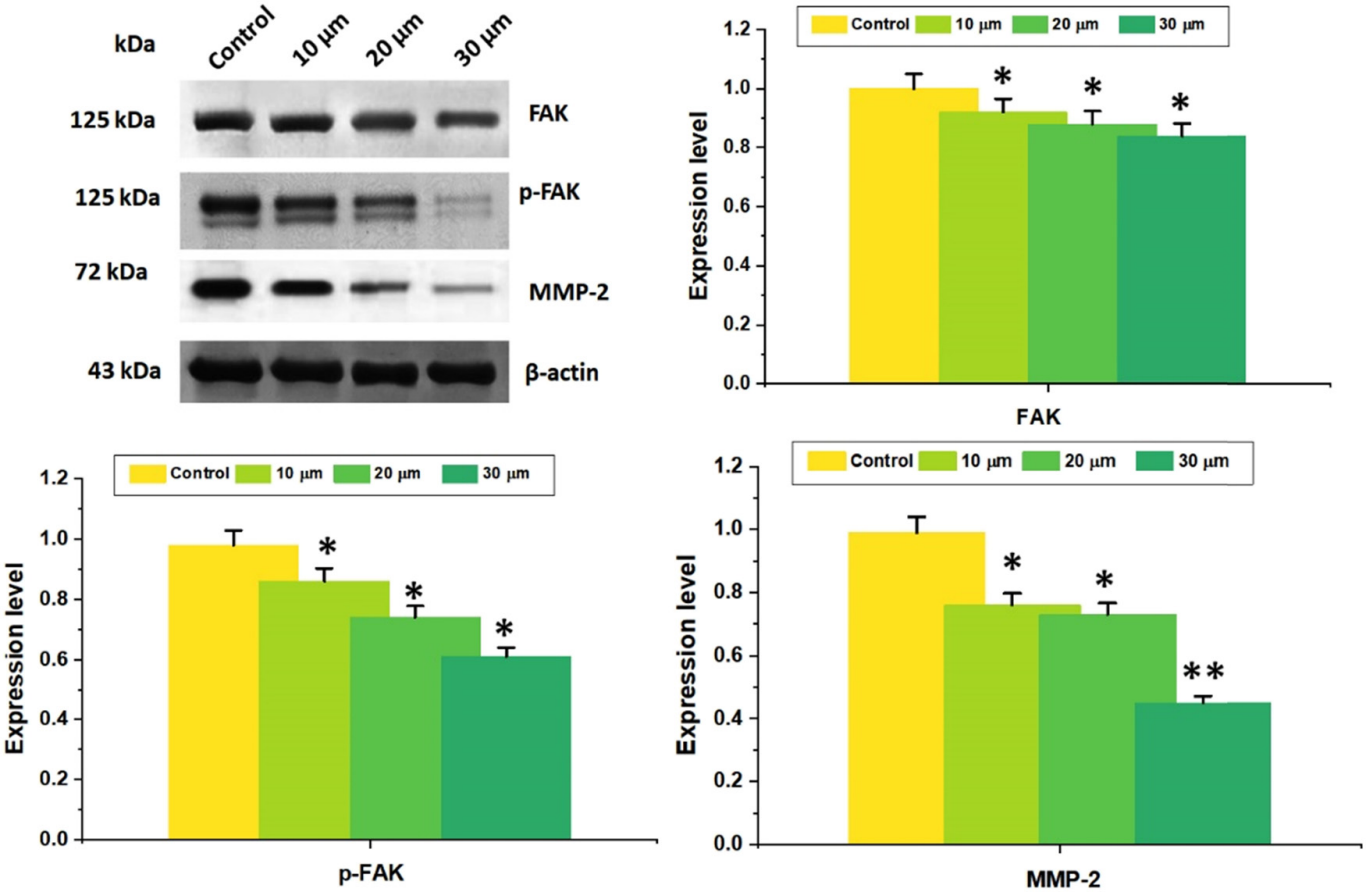
Western blotting technique was used to assess the levels of p-FAK (Tyr397) and MMP-2 in A549 cells after they were treated with thecombination of DN and OA (10, 20, 30 µM) for 24 h. As a loading control, β-Actin was employed. Quantitative evaluation of FAK, p-FAK (Tyr397), and MMP-2 protein expression ratios was performed. Values correspond to mean ± SD commencing three replicates. The mean value ± SD is shown, *P < 0.05, **P < 0.001.

To assess the anti-metastatic effect of the combo of DN + OA, the presence of tumor invasion and metastasis indicators, such as FAK, p-FAK (Tyr397), and MMP-2, was found (Figure 11a). The findings demonstrated that the DN + OA combination decreased MMP-2 expression and FAK phosphorylation in accordance with dose (Figure 11b). As a result, DN + OA combined with A549 cells suppressed cell migration. The simultaneous treatment of DN and OA on A549 cells has, therefore, simultaneously diminished cell proliferation while stimulating apoptosis by blocking the FAK/STAT3 signaling pathway, indicating that the current combination might represent an attractive prospective therapeutic strategy for the management of lung cancer.

### 3.13 Combination of DN and OA on the apoptosis quantification in A549 cells

**Figure 12:**
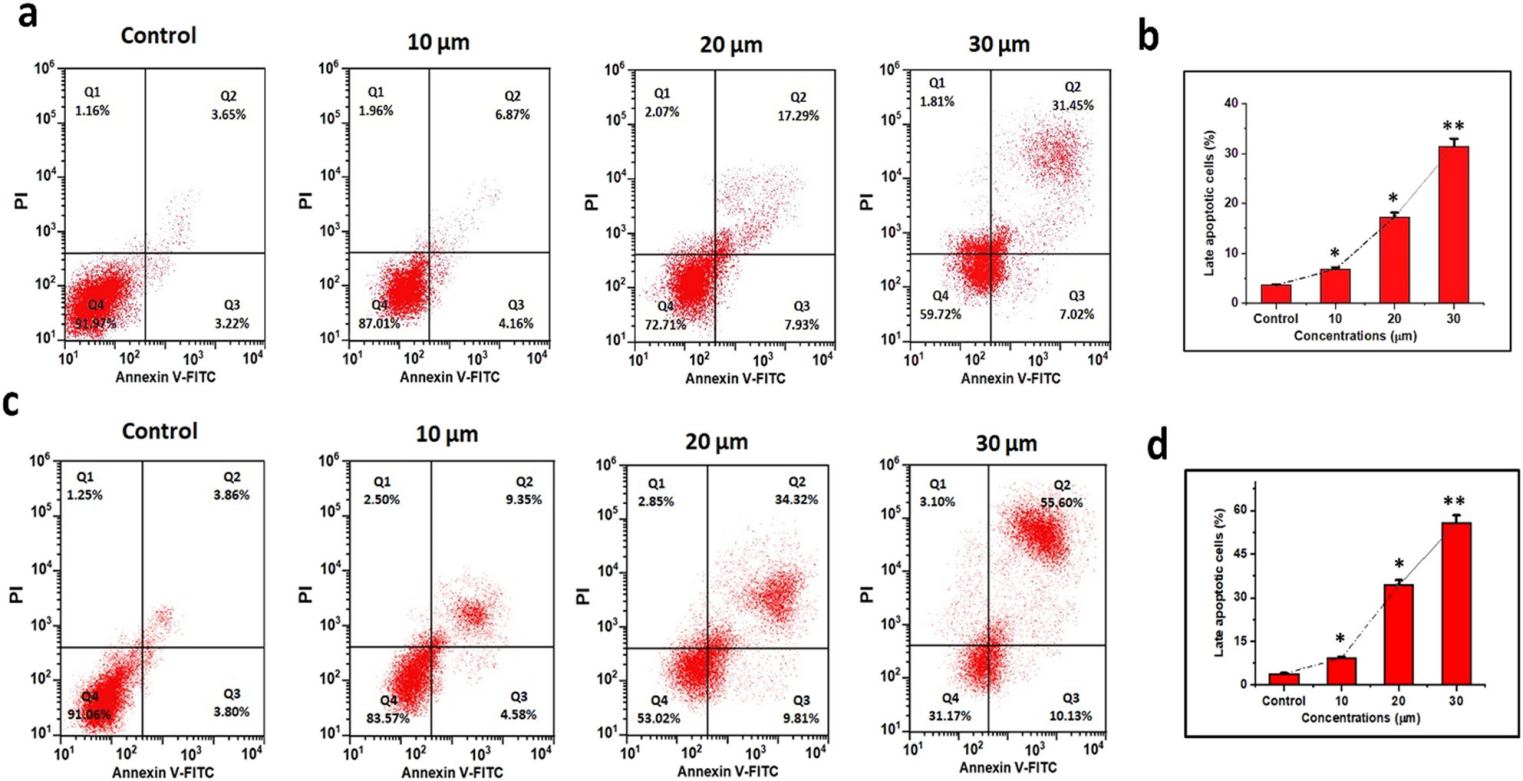
In A549 cells, the simultaneous administration of DN and OA caused apoptosis. (a, c) A549 cells were treated with varying concentrations of DN and OA (10, 20, and 30 µM) for 24 and 48 h, respectively. After that, the cells were then stained with PI and Annexin V-FITC and examined using a flow cytometer. (b, d) Statistical evaluation of tests for cell apoptosis. The mean value ± SD is shown, *P < 0.05, **P < 0.001.

Figure 12. shows the FITC-labeled annexin V and PI are two marker dyes used in flow cytometry to investigate the effects of DN and OA over a 24 and 48 h, respectively. While dead and late apoptotic cells bind to both annexin V and PI, early apoptotic cells can be labeled with annexin V dye. It is possible to discriminate between living and dead growth cells using these two colors. According to the results, only 5% of untreated A549 cells showed necrotic and apoptotic activity. DN and OA caused an increase in the proportion of apoptotic cells (Figure 12). Findings showed significant differences between the control group and the A549 cells treated with every combination of DN and OA. For DN and OA (10, 20, 30 µM/mL), the corresponding rates of apoptotic cells were 31.45% for 24 h and 55.60% for 48 h. Based on our investigation, the number of cells in the Q2 and Q3 phases of apoptosis grew in response to the combination treatment with DN + OA and A549. Treatment of A549 cells with the addition of DN and OA was found to elevate the proportion of apoptotic cells in a dose-dependent way.

## 4 Discussion

Lung carcinoma is among the most prevalent kinds of invasive cancer in the globe, having the highest mortality, and NSCLC is the most frequently diagnosed form of lung cancer [16]. While several chemotherapeutic drugs are frequently utilized for treating NSCLC. However, their clinical applicability is restricted due to their resistance to the recurrence of SCLC [17]. According to Byers and Rudin [18], the advancement of novel drugs is imperative to enhance the eradication rates of lung cancer due to its elevated recurrence rate, swift growth of treatment resistance, and inadequate effectiveness of existing therapies following recurrence.

Many different combinations are being tested for cancer treatment to treat the disease more effectively [19]; combo anticancer medications and natural substances are one such combination therapy that is being elucidated [20,21]. Plant-based remedies are not only used in East Asian traditional herbal medicine, such as that practiced in Korea, China, Europe, and America, they are also actively researched and sold as botanical pharmaceuticals. Interest in using natural goods has grown as a result of the slow progress being made in the usage of chemicals [22,23]. There are still certain obstacles to overcome in studying and developing natural ingredients as cancer treatments, notably for lung cancer [24,25]. Consequently, it is imperative to evaluate their current state by categorizing combination therapy for lung carcinoma based on the natural components they include. Clinical trials on the usage of natural remedies in cancer patients have, up to the present, primarily addressed managing the side effects or unfavorable effects of anticancer drugs on cancer patients instead of treating the illness itself. Clinical trials examining the anticancer activity of natural items are necessary to promote their usage as anticancer drugs. Therefore, in the present investigation, we found that combinations of natural drugs involving DN and OA inhibited lung cancer cell growth and induced cell death, leading to a realistic therapeutic efficacy against the illness.

Our most intriguing result proved that DN, OA, and their combined use significantly slowed the growth of A549 cells by inducing apoptosis in those cells. Numerous studies have demonstrated the anticancer properties of either OA or DN against cancer [3,26–32]. Currently, no research examines the synergistic effects of OA and DN against A549 lung cancer. Combining DN with OA caused A549 cells to undergo apoptosis, improve cytotoxicity, induce cell cycle arrest, and reduce colony formation and migration.

To cause cell death, most commercially available chemotherapy drugs have been demonstrated to increase intracellular ROS levels. For instance, cellular ROS production is lower in taxanes than in anthracyclines and platinum coordination combinations [33–35]. In the same direction, we found in our investigation that DN + OA causes ROS in A549 cells. Moreover, one mechanism by which DN with OA exerts a therapeutic benefit is the generation of ROS in cancer cells. The fluorescent intensities of DN and OA could potentially be integrated to determine the amounts of ROS within the cell. The ROS experiment’s findings indicate that A549 cells co-treated with DN and OA produce higher ROS than the untreated group.

To attempt to clarify how the DN + OA combo works on mitochondria, we measured MMP using Rh-123 dye as a marker. In A549 cells, we found that the combined use of DN + OA therapy resulted in a notable decrease in MMP. Subsequently, it has been documented that both DN and OA cause ROS-mediated mitochondrial destruction, which in turn causes apoptosis [32,35].

Numerous treatments with chemotherapy and phytochemicals have been found to target the trigger of apoptosis and cell cycle arrest as viable targets for therapy [36]. In the present investigation, it is discovered that the combination applications of DN + OA possess the processes of apoptotic cell death in lung carcinoma. Lung carcinoma cells treated with DN and OA demonstrated a notable quantity of blue fluorescence, but control calls exhibited very little blue fluorescence, as determined by DAPI staining. These results revealed that DN + OA considerably increased the level of nucleus disruption in A549 cells. The apoptotic responses of the combined use of DN + OA toward A549 cells were examined using the AO/EtBr double staining technique. Because the red magnitude fluorescence of the combination-treated cells was higher than that of the untreated cells, it was evident that the simultaneous usage of DN + OA enhanced apoptosis in the lung carcinoma cell line. Earlier investigations have demonstrated the effectiveness of luteolin and asiatic acid in hindering the development of cervical cancer [37]. On the other hand, DN leads to apoptosis and cell cycle arrest in breast cancer by controlling the NF-κB and MAPK signaling channels [28]. In addition to its dual effects on PPARγ upregulation and PGRMC1/2 expression pattern reversal, OA prevents ovarian cancer cells from migrating and causes them to undergo apoptosis [30].

Prior research has indicated that DN can cause G2/M cell cycle arrest in leukemia cancer cell lines by raising Cdk1’s phosphorylation at Tyr15 and the protein expression levels of Cdk inhibitors [38,39]. According to Clemente-Soto et al. [40], quercetin activated p53, which in turn caused apoptosis in the HeLa and SiHa cell lines and increased the protein expression level of p21WAF1/Cip1. Moreover, OA inhibits the development of cancerous cells in the colon and causes G2/M cell cycle arrest [41]. In the current investigation, it was shown that the simultaneous administration of DN and OA may cause G2/M cell cycle arrest in cancerous lung cell lines A549, and the cause of this arrest was linked to the suppression of several important cell cycle-related genes.

According to reports, the FAK/STAT3 signaling mechanism is important for cancer cells’ movement, invasion, and survivability [42]. Accelerated proliferation and spread of cancer cells can be caused by improper STAT3 activation [43,44]. According to Mohrherr et al. [45], preventing STAT3 activation declines cell growth, demonstrating the critical role that STAT3 plays as an avenue for lung cancer therapy. This investigation showed that the influence of dose reduction of STAT3 phosphorylation was achieved by using the combination of DN + OA. Additionally, the DN + OA combo controlled the expression of multiple STAT3 downstream proteins, such as pro-proliferative protein (cyclin D1), pro-apoptotic protein (Bax), and anti-apoptotic protein (Bcl-2). In addition to inhibiting the STAT3 signaling system, the simultaneous administration of DN and OA may stimulate the apoptotic pathway. Consequently, the capacity of the combined effect of DN + OA to cause apoptosis may be associated with the increasing activity of Bax and a reduction inf Bcl-2 and cyclin D1 expression rates.

Furthermore, via triggering MMPs, FAK is essential for tumor invasion [46]. Tumor angiogenesis is mainly caused by MMP-2. According to Cho et al. [47], and Palmisano and Itoh [48], blocking MMPs typically precludes the development of additional capillaries, which in turn restricts the migration and invasion of tumor cells. The present investigation demonstrated that the simultaneous use of DN + OA significantly suppressed FAK phosphorylation and downregulated MMP-2, augmenting its capacity to impede A549 cell migration. Consequently, signal transduction linked to invasion and metastasis was likewise inhibited by the DN + OA therapy combo.

## 5 Conclusion

The present investigation was designed to ascertain the synergistic effects of DN and OA in regulating the growth of A549 cells. The investigation’s principal discovery is that the simultaneous use of DN and OA induces apoptosis and inhibits A549 cell growth. Additional studies examine how the combo of DN and OA affects additional apoptotic and anti-apoptotic mechanisms in lung cancer. By inhibiting the FAK/MMP-2 signaling process, the combined use of DN + OA exhibited anti-metastatic impacts and anti-proliferative activities via inhibiting the STAT3 signaling pathway. Furthermore, by limiting the phosphorylation of STAT3 and controlling the expression of STAT3 downstream proteins, such as Bcl-2 and cyclin D1, the combined effect of DN + OA impeded the growth of cells. In the meantime, by decreasing MMP-2 expression and blocking FAK phosphorylation, the combined use of N and OA prevented cell migration. The results of this study suggest that a combo of DN and OA may one day be employed as a novel and promising agent in the fight against lung carcinoma.
